# Immune Checkpoint Inhibitor-Induced Myositis/Myasthenia Gravis Overlap

**DOI:** 10.7759/cureus.49007

**Published:** 2023-11-18

**Authors:** Caroline Gosser, Anas Al Bawaliz, Waled Bahaj, Jason Chesney, Smita Ranjan

**Affiliations:** 1 Internal Medicine, University of Louisville School of Medicine, Louisville, USA; 2 Hematology and Medical Oncology, University of Louisville Hospital, Louisville, USA

**Keywords:** rituximab, myasthenia gravis, myositis, immune checkpoint inhibitor, oncology

## Abstract

Immune checkpoint inhibitors (ICIs) have considerably changed the management of several malignancies. Although these agents transformed the scope of management in oncology and proved long-term efficacy, they have been associated with numerous autoimmune-related adverse events.

We presented a case of a 61-year-old male with a history of non-small cell lung cancer (NSCLC) who presented with respiratory failure requiring mechanical ventilation. He was discharged with a working diagnosis of myasthenia gravis crisis secondary to the use of pembrolizumab. On further evaluation, he was found to possibly have pembrolizumab-induced myositis. He was treated with plasmapheresis, methylprednisolone, and rituximab and achieved significant improvement.

Pembrolizumab, a monoclonal antibody, is an ICI that targets programmed death protein 1 (PD-1), thereby blocking the interaction between PD-1 and PDL-1, leading to an enhancement of T-cell mediated immune response against tumor cells. Pembrolizumab has been used to treat a variety of malignancies including melanoma, NSCLC, and other solid tumors. Though ICIs have revolutionized the field of oncology, they should be used with caution. ICIs can cause immune-related adverse events (irAEs), including myasthenia gravis and myositis. Diagnosing irAEs is challenging due to their nonspecific presentations and lack of antibody markers. Therefore, patients and clinicians should be aware of irAEs in order to initiate timely intervention.

## Introduction

Immune checkpoint inhibitors (ICIs) are monoclonal antibodies that are biological response modifiers. They have been utilized in cancer treatments to impair tumor cell function and growth, deliver toxic agents to tumors, and enhance host cell-mediated immunity. Pembrolizumab is an IgG monoclonal antibody that inhibits programmed cell death-1 (PD-1) receptors. When PD-1 binds to its ligand PD-L1, on an immune cell, cell-mediated toxicity is prevented, and tumor cells evade the immune system [1]. Inhibition of this process using pembrolizumab results in upregulation of cytotoxic T-cells, thus reducing the tumor burden. Pembrolizumab has been used for the first-line treatment of primary cancers such as non-small cell lung cancer (NSCLC) and a variety of metastatic cancers, including melanoma, head and neck squamous cell cancer, advanced urothelial cancer, colorectal cancer, cervical cancer, and others [2]. The wide adoption of pembrolizumab in cancer treatment has provided more information about its safety profile. Pembrolizumab has been linked to dermatological, muscular, pulmonary, gastrointestinal, neurological, cardiac, and endocrine complications [3-8]. Most ICI side effects are reversible with medical treatment, mainly steroids, but some lead to permanent disease.

## Case presentation

A 61-year-old Caucasian male presented to the emergency department in August of 2022 with altered mental status and respiratory failure. He has a past medical history of metastatic non-small cell lung adenocarcinoma, diagnosed in February of 2021, and had recently completed his fourth cycle of Carboplatin, Pemetrexed, and Pembrolizumab. He received 200 mg of Pembrolizumab intravenously every three weeks. In May of 2022, he started developing symptoms of progressive bilateral ptosis, dysphagia, dysarthria, weakness in his tongue, and severe fatigue. He was seen by a neurologist in June of 2022, who diagnosed him with myasthenia gravis. He was started on pyridostigmine 60 mg three times daily and prednisone taper. The patient did not have an antibody study at that time. After the myasthenia gravis diagnosis the pembrolizumab was held. In August of 2022, he felt his condition had improved, and he stopped taking his prednisone for three days. He quickly deteriorated and was found shortly thereafter unresponsive at home. Upon arrival at the emergency department, a head CT scan and chest x-ray were performed due to altered mental status and respiratory distress. The head CT was negative for an acute intracranial abnormality. Pneumonia and other infectious respiratory illnesses were ruled out due to a lack of radiographic and relevant clinical findings. Guillain-Barre syndrome was considered unlikely due to the lack of clinical supporting evidence. Other markers were negative, including CPK, ESR, CRP, and ANA. Thus, the patient was presumptively diagnosed with myasthenia crisis secondary to immune therapy with pembrolizumab. His acetylcholine receptor antibody was 0.30 nmol/L (positive acetylcholine receptor antibody test requires 0.50 nmol/L).

Upon presentation to the emergency department, the patient was intubated and sedated due to his severe hypoxia and inability to protect his airway. Two days later, he was started on plasmapheresis (PLEX) and received five treatments. He was also started on methylprednisolone 2mg/kg/day. An attempt to restart pyridostigmine prescribed in May of 2022 due to lid lag and dysphagia was discontinued due to ineffectiveness and side effects such as nausea and muscle cramping. The patient's condition improved, and on day 2 of admission, he was able to be extubated. The patient was discharged seven days following admission. Upon discharge, he was prescribed 1.50 mg/kg of prednisone daily and continued with PLEX treatment weekly. The patient had a relapse, resulting in another admission to the hospital nine days after being discharged. Since that admission, the patient began weekly outpatient PLEX treatments and a tapering regimen of prednisone. In early fall of 2022, the patient's chemotherapy, consisting of a pemetrexed single agent, was restarted (the carboplatin regimen was completed in four cycles at the onset of treatment). Due to the severe nature of the patient's reaction to Pembrolizumab, it was not restarted after discontinuing it in June of 2022. No replacement was prescribed in place of Pembrolizumab for the patient's NSCLC. He did not undergo next-generation sequencing for targeted therapy. In the fall of 2022, he endorsed mild improvement of symptoms but still had significant muscle weakness.

**Figure 1:**
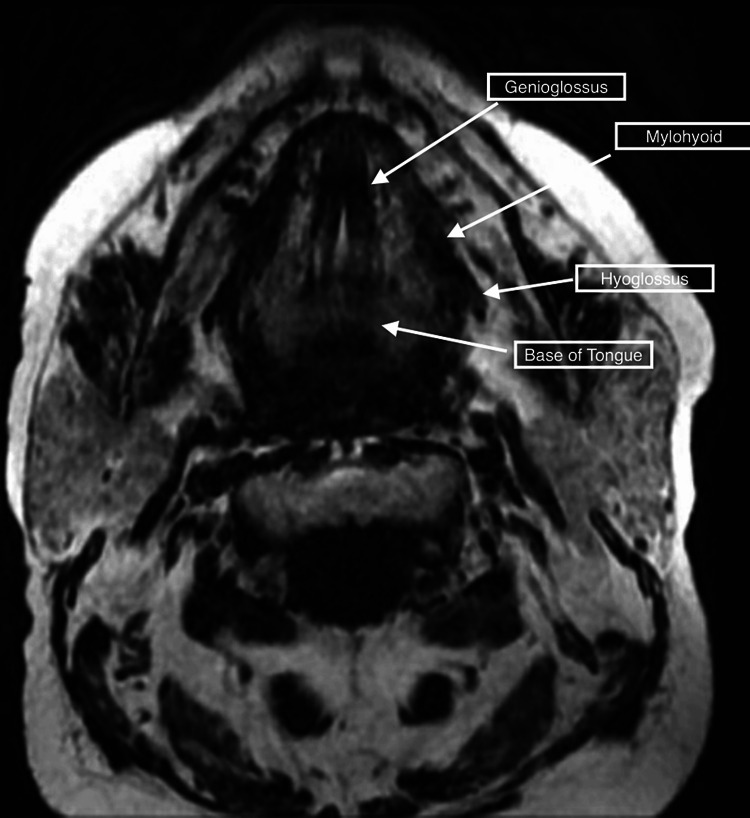
Axial facial MRI without contrast demonstrating masseter fatty infiltration. Tongue root and oral tongue musculature atrophy can also be seen.

**Figure 2:**
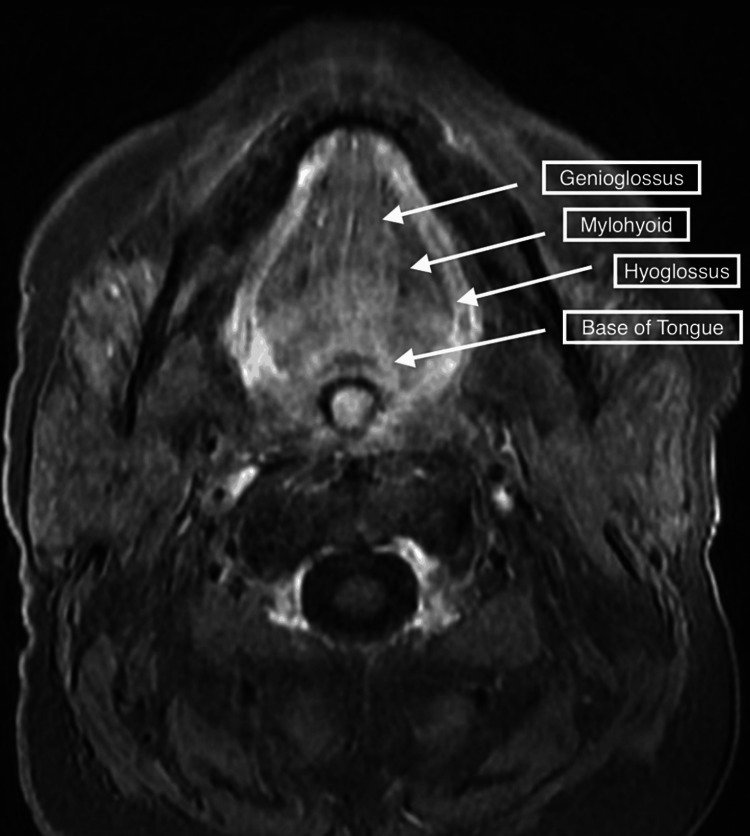
Axial gadolinium-enhanced T1-weighted image demonstrating tongue root and oral tongue muscular enhancement likely indicating acute myopathy or denervation.

**Figure 3:**
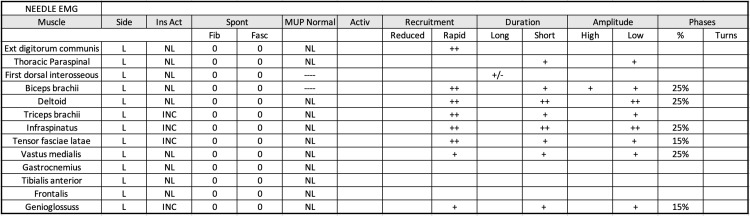
Needle EMG results showed low-amplitude and complex motor unit potentials with early recruitment in the tongue and proximal and axial muscles. This is indicative of a defect of neuromuscular transmission but likely secondary to primary myopathic process.

Since the patient continued to have significant weakness with the above treatment, he was referred to an outside hospital in late fall of 2022. Further myositis work-up including Anti-MDA5 Ab (CADM-140); Anti-TIF-1gamma Ab; Anti-SAE1 Ab, IgG; Anti-SRP Ab; Anti-U3 RNP (Fibrillarin Ab); Anti-U2 RNP Ab; Anti-Mi-2 Ab; Anti-PL-7 Ab; Anti-PL-12 Ab; Anti-OJ Ab; Anti-Jo-1 Ab; an Anti-U1 RNP Ab were all negative. A myasthenia gravis panel was also negative during this visit. A facial MRI was conducted, which showed signs of muscle fatty infiltration. Atrophy in the tongue root and oral tongue muscles was also depicted (Figure 1). Tongue root and oral tongue muscular enhancement in a contrast MRI were presumed to indicate acute myopathy (Figure 2). A biopsy of the patient's right infraspinatus muscle was conducted. The biopsy was stained with Ulex europaeus agglutinin I and the C5b9 complement membrane attack complex (MAC), MHC-1, and MxA. The biopsy revealed no capillary depletion with granular complement deposition on the sarcolemmal membrane of rare non-necrotic fibers but not on capillaries which suggested an underlying autoimmune process. Regenerating fibers were also observed on the muscle stain indicating a myopathy. Lastly, some fibers were atrophic suggesting denervation atrophy with associated reinnervation. A needle EMG showed low-amplitude and complex motor unit potentials with early recruitment in the tongue and proximal and axial muscles, indicating the patient's condition was likely myopathic, not neurogenic (Figure 3). Thus, the patient was diagnosed with inflammatory myositis secondary to pembrolizumab without evidence of a neuromuscular junction disorder. However, myasthenia could not be definitively ruled out.

The providers at the outside facility suggested the patient use the monoclonal antibody Tocilizumab. However, the patient's financial status did not allow for the use of Tocilizumab. Thus, the alternative suggestion was rituximab in an attempt to lower the risk of long-term steroid and PLEX treatment and the patient's inadequate response to the initial treatments. The patient began with weekly rituximab doses for four weeks, then transitioned to monthly doses. Plasmapheresis was discontinued upon initiation of rituximab. After beginning rituximab, the patient did have an improvement in the strength of facial muscles as he was able to keep his eyes open for extended time periods. The patient also had significant improvement of his lower extremity weakness and he could ambulate on his own for short distances.

## Discussion

The antibody PD-1 has a negative regulatory role in the immune system, preventing the formation of self-reactive T-cells and promoting the development of T-regulatory cells [9]. Thus, eliminating PD-1 or PD-L1 can result in uncontrolled T-cell responses leading to autoimmunity.

Tumor cells generate an immune response initially but then can suppress the response due to the expression of PD-L1. The tumor microenvironment also provokes the expression of PD-1 in T cells. PD-1 expression is upregulated in cells when exposed to antigens by various cytokines, including interferon-γ, interleukin (IL)-2, IL-7, IL-15, and IL-21 [10]. The interaction of PD-L1 on tumor cells and PD-1 on T lymphocytes causes apoptosis of T cells, inhibition of cytokine production, and immunosuppression. This process allows for the growth of the tumor cell.

ICIs are relatively new treatment therapies for various cancers, including NSCLCs. Pembrolizumab increases tumor immunogenicity by inhibiting programmed cell death-1 (PD-1) activity by binding to the PD-1 receptor on T-cells to block PD-1 ligands (PD-L1 and PD-L2) from binding. This leads to a reduction of the tumor burden and an increase in the immune response throughout the body. Therefore, ICIs can result in various autoimmune responses and adverse effects.

ICI myositis is a rare but severe side effect. One retrospective cohort study conducted at the University of Texas MD Anderson found that 36 out of 9,088 patients developed myositis. Of those 36 patients, nineteen experienced overlap with other diseases (five with myocarditis, five with myasthenia gravis, and nine with both) [11]. The mean period to the onset of ICI-induced myositis from the initiation of ICI was four weeks [12]. Patients can have a variable presentation ranging from moderate myalgia and generalized muscle weakness to dysphagia, dysarthria, and dysphonia [11]. Our patient's myositis appeared to be preferentially attacking oculobulbar muscles, producing a clinical presentation similar to myasthenia gravis. Most patients also have elevated CK levels ranging from the hundreds to over 10,000 U/L (normal levels are 25-160 U/L) [13]. However, our patient's CK levels were within a normal range. Myositis serum antibody panels can also be conducted for patients, but when done in the setting of ICI-induced myositis, those autoantibodies are likely to be negative.

A muscle biopsy is currently the most definitive test to diagnose myositis. The biopsy might show overexpression of MHC class 1, abnormal C5b9 deposits on muscular membranes, numerous necrotizing muscular fibers, and endomysial CD8+ T-cell-predominant infiltrates [14]. The management of patients with ICI-induced myositis is to begin high-dose steroids (1-2 mg/kg) and taper as the patient demonstrates improvement. Those patients experiencing myasthenia-like symptoms can also benefit from IVIG. Some patients may have to discontinue their ICI. If patients continue to use their ICI, CK levels should be regularly monitored, as well as any myositis-related symptoms.

Other treatments are being investigated for refractory ICI-induced disorders as the current treatments listed above have significant side effects. Tocilizumab is an IL-6 receptor antibody that blocks the signaling of the cytokine IL-6. IL-6 is an essential component of acute and chronic inflammation. Furthermore, IL-17A-expressing CD4+ T cells have been found to be a factor in autoimmune inflammation refractory to steroid treatment [15]. In model studies, IL-6 blockade with anti-IL-6 receptor antibody lessened the severity of C protein-induced myositis [16]. Thus, Tocilizumab has a high probability of improving or slowing the progression of ICI myositis. In fact, tocilizumab has been used successfully in other cases of ICI disease, for example, myocarditis [17].

Our patient was trialed on rituximab, a CD-20 monoclonal antibody, in order to avoid the adverse outcomes of high-dose steroids and PLEX treatments. Cruz et al. proposed that rituximab could be beneficial in ICI-induced autoimmune disorders due to the suppression of B-cell-driven autoantibody formation induced by loss of function of Treg cells [18]. One review found that patients with inflammatory myopathies treated with rituximab had a 78.3% response rate [19]. Those with autoantibody-positive myopathies responded significantly better to rituximab treatment than those without autoantibodies specific to myositis [19].

## Conclusions

We have described a case of inflammatory myositis with possible overlap with myasthenia gravis induced by the monoclonal antibody pembrolizumab. While treatment with high-dose steroids and plasmapheresis may provide help, adjunct therapies may still be needed in refractory cases. Treatment with rituximab led to meaningful clinical improvement in our described case. Although cases of myositis induced by ICIs have been described in the literature, no clear protocols have been established for treating these patients beyond the dosage of high-dose steroids and PLEX, which carry long-term risks. Therefore, further studies need to be devised on treating this immune-related adverse event and evaluating the role of rituximab and other adjunct therapies.
